# Supplementary material for: Incidence Rate and Determinants of Recurrent Cholesteatoma Following Surgical Management: A Systematic Review, Subgroup, and Meta-Regression Analysis
Source: Biomedicines. 2025 Oct 14;13(10):2506. doi: 10.3390/biomedicines13102506 (PMC12562222; doi:10.3390/biomedicines13102506)
Supplement: Supplementary file 1 [file biomedicines-13-02506-s001.zip › biomedicines-3836107-supplementary.pdf]

Table S1. The search syntax employed in the database search of this review

Database	No	Search Query	Results
**PubMed**	#1	Cholesteatoma[tiab] OR "Cholesteatoma"[Mesh]	8627
	#2	Recurrence[tiab] OR recurrent[tiab] OR residual[tiab] OR "Recurrence"[Mesh] OR recidivism[tiab]	1009119
	#3	Rate[tiab] OR prevalence[tiab] OR incidence[tiab] OR "risk factor*"[tiab] OR predict*[tiab] OR determinant*[tiab] OR "relative risk" OR "odds ratio" OR regression OR hazard* OR "Risk Factors"[Mesh]	7481319
	#4	Surgery OR surgical OR operation OR mastoidectomy OR tympanoplasty OR "Mastoidectomy"[Mesh] OR "Tympanoplasty"[Mesh]	6848082
	#5	#1 AND #2 AND #3 AND #4	645
**Scopus**	#1	TITLE-ABS-KEY (Cholesteatoma)	11705
	#2	TITLE-ABS-KEY (Recurrence) OR TITLE-ABS-KEY (recurrent) OR TITLE-ABS-KEY (residual) OR TITLE-ABS-KEY (recidivism)	1995838
	#3	TITLE-ABS-KEY (Rate) OR TITLE-ABS-KEY (prevalence) OR TITLE-ABS-KEY (incidence) OR TITLE-ABS-KEY ("risk factor*") OR TITLE-ABS-KEY (predict*) OR TITLE-ABS-KEY (determinant*) OR ALL ("relative risk") OR ALL ("odds ratio") OR ALL (regression) OR ALL (hazard*)	20609642
	#4	ALL (Surgery) OR ALL (surgical) OR ALL (operation) OR ALL (mastoidectomy) OR ALL (tympanoplasty)	14209574
	#5	#1 AND #2 AND #3 AND #4	974
**Web of Science**	#1	AB=Cholesteatoma	4156
	#2	AB=Recurrence OR AB=recurrent OR AB=residual OR AB=recidivism	1179456
	#3	AB=Rate OR AB=prevalence OR AB=incidence OR AB="risk factor*" OR AB=predict* OR AB=determinant* OR ALL="relative risk" OR ALL="odds ratio" OR ALL=regression OR ALL=hazard*	12670139
	#4	ALL=Surgery OR ALL=surgical OR ALL=operation OR ALL=mastoidectomy OR ALL=tympanoplasty	6266924
	#5	#1 AND #2 AND #3 AND #4	550
**CENTRAL (Cochrane Registry of Randomized Trials**	#1	Cholesteatoma	194
	#2	Recurrence OR recurrent OR residual OR recidivism	116123
	#3	Rate OR prevalence OR incidence OR "risk factor" OR "risk factors" OR predict* OR determinant* OR "relative risk" OR "odds ratio" OR regression OR hazard*	724712
	#4	Surgery OR surgical OR operation OR mastoidectomy OR tympanoplasty	370997
	#5	#1 AND #2 AND #3 AND #4	26
**Google Scholar**	With all of the words	Cholesteatoma recurrence	-
	With the	-	-

	exact phrase		
	With at least one of the words	predict risk factor determinant regression hazard odds	-
	Total	As per recent recommendations, only the first 200 records were selected	200

Table S2. The definition criteria of recurrent cholesteatoma in included studies

Author (YOP)	Definition
**Abdullah et al. (2013)**	-
**Abraham et al. (2022)**	Recurrent disease was defined as the presence of cholesteatoma detected after 6 months, with previous documented disease-free visits.
**Adriaansens et al. (2022)**	-
**Alam (2022)**	The recurrence was diagnosed as patients developed foul smelling discharge and oto-microscopic examination revealed presence of cholesteatoma in the follow-up period
**Alicandri-Ciufelli et al. (2016)**	Recurrences (defined as non–self-cleaning re-retraction of the attic requiring surgery) and residual (defined as insufficient primary resection of the epidermal matrix, presenting as cholesteatoma in absence of re-retraction of the tympanic membrane) were noted in the database.
**Alvarez et al. (2011)**	-
**Arias Marzán et al. (2023)**	-
**Aslan Felek et al. (2009)**	-
**Bakaj et al. (2016)**	-
**Barakate (2008)**	-
**Chamoli et al. (2018)**	-
**Cheng et al. (2023)**	-
**Cho et al. (2016)**	We defined recurrent cholesteatoma ma as a whitish mass found beyond the tympanic membrane or as pathologically confirmed cholesteatoma after revision surgery because of otologic symptoms such as otalgia and otorrhea during the follow-up period.
**Choi et al. (2010)**	-
**Crowson et al. (2016)**	Recurrent cholesteatoma was defined as having found cholesteatoma in a new retraction pocket.
**Danesi et al. (2016)**	-
**Darrouzet et al. (2000)**	-
**Das et al. (2019)**	The definitive proof of residual/recurrent cholesteatoma and understanding the pathophysiology of chronic draining ear could be realized only at surgical exploration, which confirms the clinical suspicion and radiologic suggestions.
**De Corso et al. (2006)**	-
**DeRowe et al. (2005)**	-
**Diom et al. (2013)**	-
**Edfeldt et al. (2012)**	-
**Edfeldt et al. (2012)**	-
**Erfurt et al. (2024)**	Recurrent disease encompasses a newly formed retraction pocket with cholesteatoma, visible by either micro-otoscopy, detected on MRI-DWI or during second look surgery.
**Ferlito et al. (2022)**	Recurrent cholesteatoma was defined as cholesteatoma developing after complete removal.
**Glikson et al. (2019)**	-

**Govil et al. (2015)**	-
**Hatano et al. (2010)**	Recurrent cholesteatoma was defined as a new cholesteatoma developing from a postoperative retraction pocket.
**Hatano et al. (2016)**	-
**Hellingman et al. (2019)**	Recurrent cholesteatoma is defined as a new cholesteatoma, developing from an unsafe, non–self-cleaning retraction pocket.
**Hou et al. (2021)**	-
**Hu et al. (2023)**	
**Ikeda et al. (2003)**	-
**Inanli et al. (2001)**	-
**James (2024)**	Recurrent cholesteatoma was defined as an accumulation of keratin debris from squamous epithelium that was in continuity with the surface of the tympanic membrane and could not be removed with micro-debridement.
**Jenks et al. (2022)**	Cholesteatoma arising from a retraction pocket that developed postoperatively was classified as recurrence.
**Killeen et al. (2019)**	Recurrent disease defined as retraction of the tympanic membrane/attic on clinical examination.
**Kim et al. (2009)**	-
**Komori et al. (2018)**	
**Komori et al. (2021)**	-
**Kuo et al. (2012)**	-
**Lazard et al. (2007)**	-
**Lee et al. (2015)**	
**Manzoor et al. (2022)**	
**Marchioni et al. (2013)**	Defined as non–self-cleaning re-retraction of the attic requiring surgery
**Marchioni et al. (2015)**	Recurrent cholesteatoma was defined as a new cholesteatoma developing from a newly formed, non-self-cleaning retraction pocket
**Minovi et al. (2014)**	-
**Mishiro et al. (2008)**	-
**Mizutari et al. (2021)**	-
**Moller et al. (2020)**	Recurrence of cholesteatoma was defined as cholesteatoma in a subsequent surgery on the same ear
**Morita et al. (2014)**	We clearly divided the recurrence into the two types; the residual cholesteatoma and the retraction cholesteatoma. Residual cholesteatoma was defined as the rest of epithelium at the previous operation, while retraction cholesteatoma was seen as deep pocket formation due to failure of ventilation that requires surgery.
**Morita et al. (2017)**	-
**Motegi et al. (2020)**	Recurrence was defined in cases where the patients re quired re-operation for recurrent cholesteatoma or the patients with tympanic membrane re-retraction, wherein, the bottom could not be observed and debris accumulation occurred, even when they did not require re-operation.
**Myers et al. (2000)**	-
**Nassif et al. (2024)**	-
**Neudert et al. (2014)**	Recurrent disease showed clinical findings like poorly visualized retraction pockets, perforations of the tympanic membrane, and/or destructions of the

	lateral attic or posterior canal wall upon preoperative microscopic examination.
**Pareschi et al. (2019)**	-
**Park et al. (2009)**	-
**Park et al. (2011)**	Recurrence was confirmed by detecting the cholesteatoma during revision surgery, regardless of the cause of the operation.
**Piras et al. (2021)**	Recurrent cholesteatoma is considered as a newly formed disease process secondary to non-self-cleansing (unsafe) retraction pocket, while residual cholesteatoma is persistence of keratinizing squamous epithelium left behind inadvertently during first-stage surgery, which has regrown into a visually identifiable cholesteatoma.
**Prasad et al. (2014)**	Recurrent cholesteatoma was de fined as a newly formed disease process secondary to a retraction pocket after second stage surgery
**Presutti et al. (2018)**	A self-cleaning retraction pocket or a non-self-cleaning retraction pocket defined as recurrent disease requiring prompt revision surgery.
**Qotb et al. (2017)**	-
**Quérat et al. (2014)**	Surgical revision was required when the cholesteatoma was difficult to eradicate during the first procedure, when audiometry performed 18 to 24 months after the operation revealed a residual air-bone gap greater than 20 dB or when otoscopy and/or CT scan suggested recurrence or residual cholesteatoma.
**Reddy et al. (2001)**	-
**Roth et al. (2013)**	-
**Roux et al. (2015)**	Recurrent cholesteatoma was defined as the redevelopment of a tympanic retraction pocket after removal of the cholesteatoma as observed on micro-otoscopic examination.
**Schraff (2006)**	We have defined recurrence as disease found at the time of second look which included disease purposely left at the time of the first operation (due to oval window involvement, dehiscent facial nerve involvement with extensive granulation tissue, etc.), as well as disease that was felt to have been fully excised but discovered at re-exploration. We did not include cases of recurrent cholesteatoma due to new retraction pockets or other causes.
**Shin et al. (2023)**	-
**Silvola (2000)**	-
**Song et al. (2019)**	-
**Sun et al. (2010)**	-
**Trinidade et al. (2015)**	-
**van Dinther et al. (2015)**	Recurrent cholesteatoma is defined as a new cholesteatoma, developing from an unsafe, non-self-cleaning retraction pocket, identified by micro-otoscopy.
**van Waegeningh et al. (2021)**	Defined as an expanding keratin accumulation inside a newly formed non-self-cleaning retraction pocket detected at routine yearly micro-otoscopy follow up.
**Vartiainen (2000)**	-
**Visvanathan et al. (2012)**	Recurrent cholesteatoma was defined as a newly formed retraction pocket extending to the attic, while residual cholesteatoma was recorded if an epithelial pearl was seen arising from a remnant of cholesteatoma matrix.

**Walker et al. (2014)**	-
**Wilson et al. (2013)**	Recurrence is defined as reformation of a cholesteatoma from retraction of the tympanic membrane.
**Wu et al. (2020)**	-
**Yamamoto et al. (2014)**	Severe retraction without a visible bottom.
**Yang et (2014)**	-
**Yung et al. (2007)**	-
**Zanetti et al. (2018)**	-

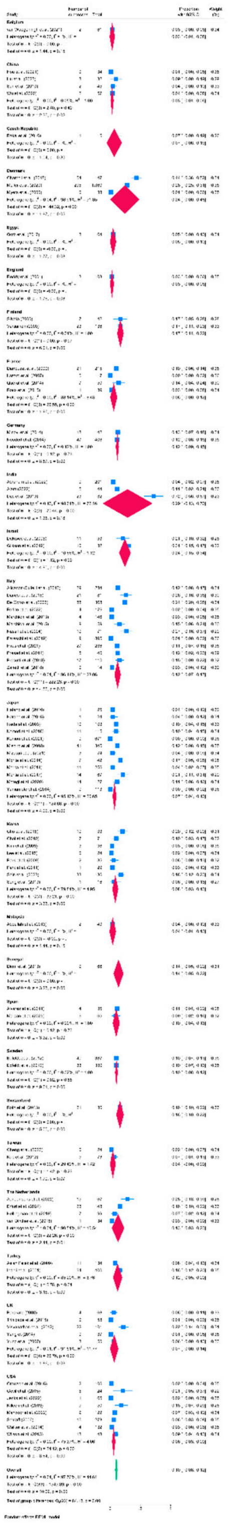

Figure S1. Forest plot showing the pooled cholesteatoma recurrence rate stratified by country of investigation

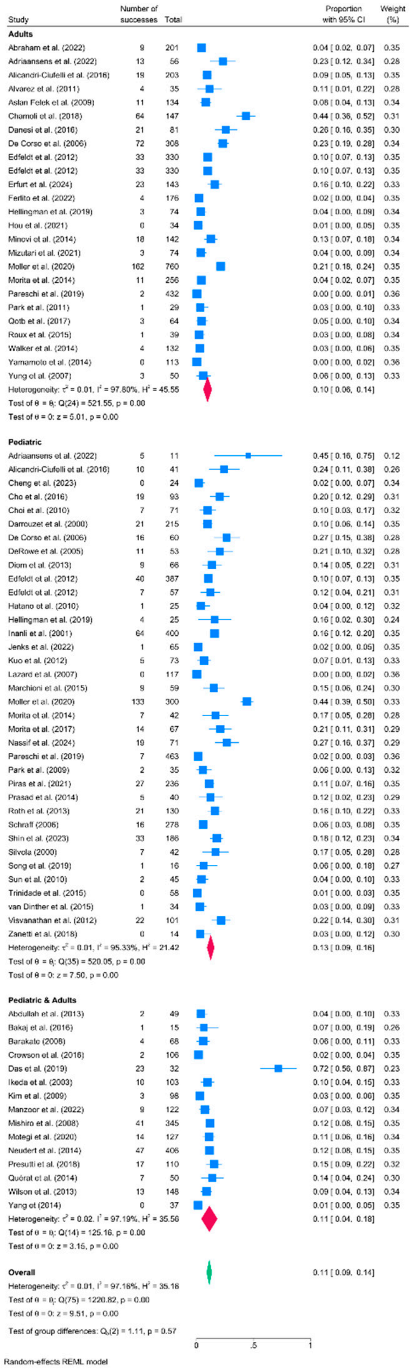

Figure S2. Forest plot showing the pooled cholesteatoma recurrence rate stratified by patients' age group

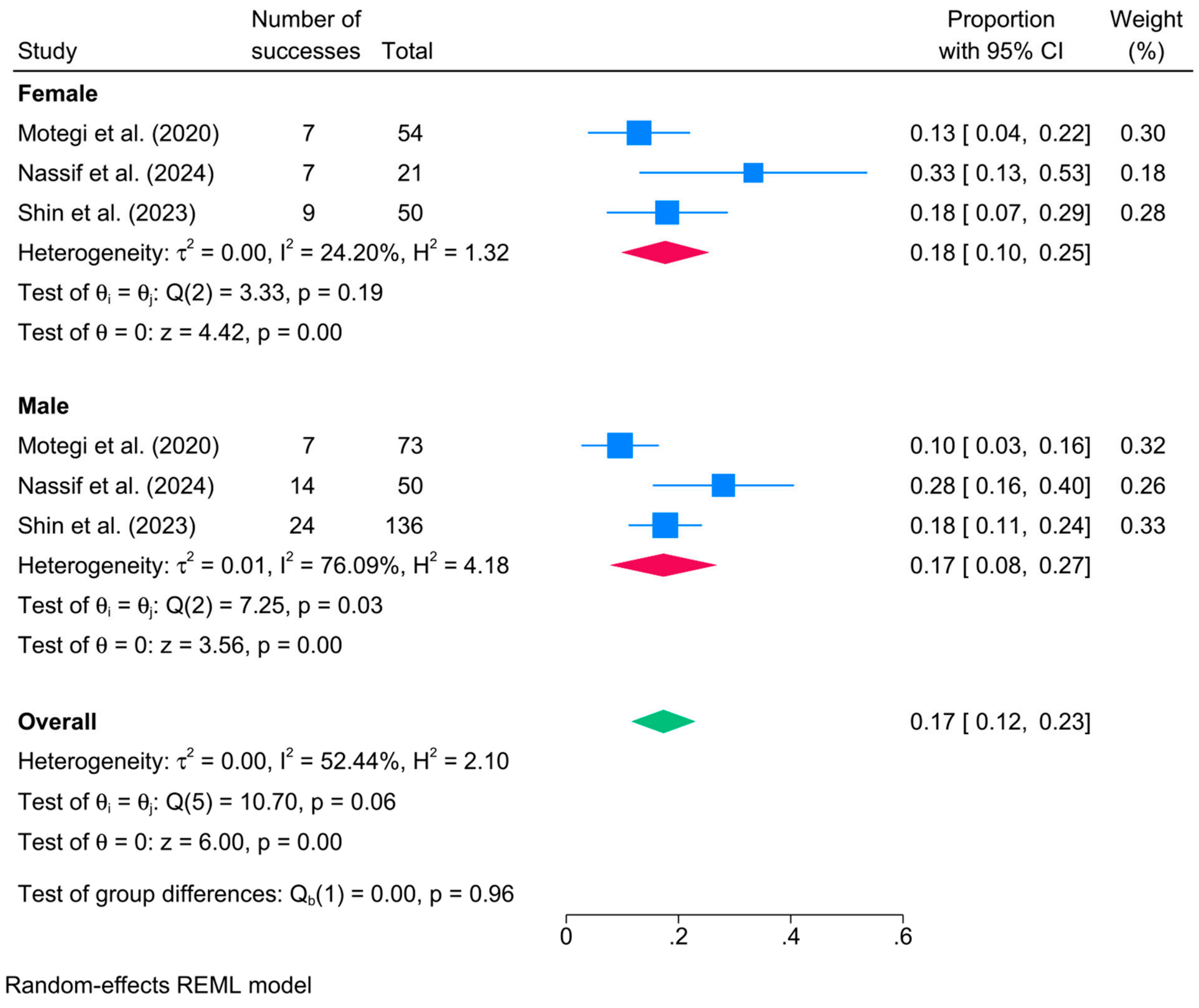

Figure S3. Forest plot showing the pooled cholesteatoma recurrence rate stratified by patients' gender

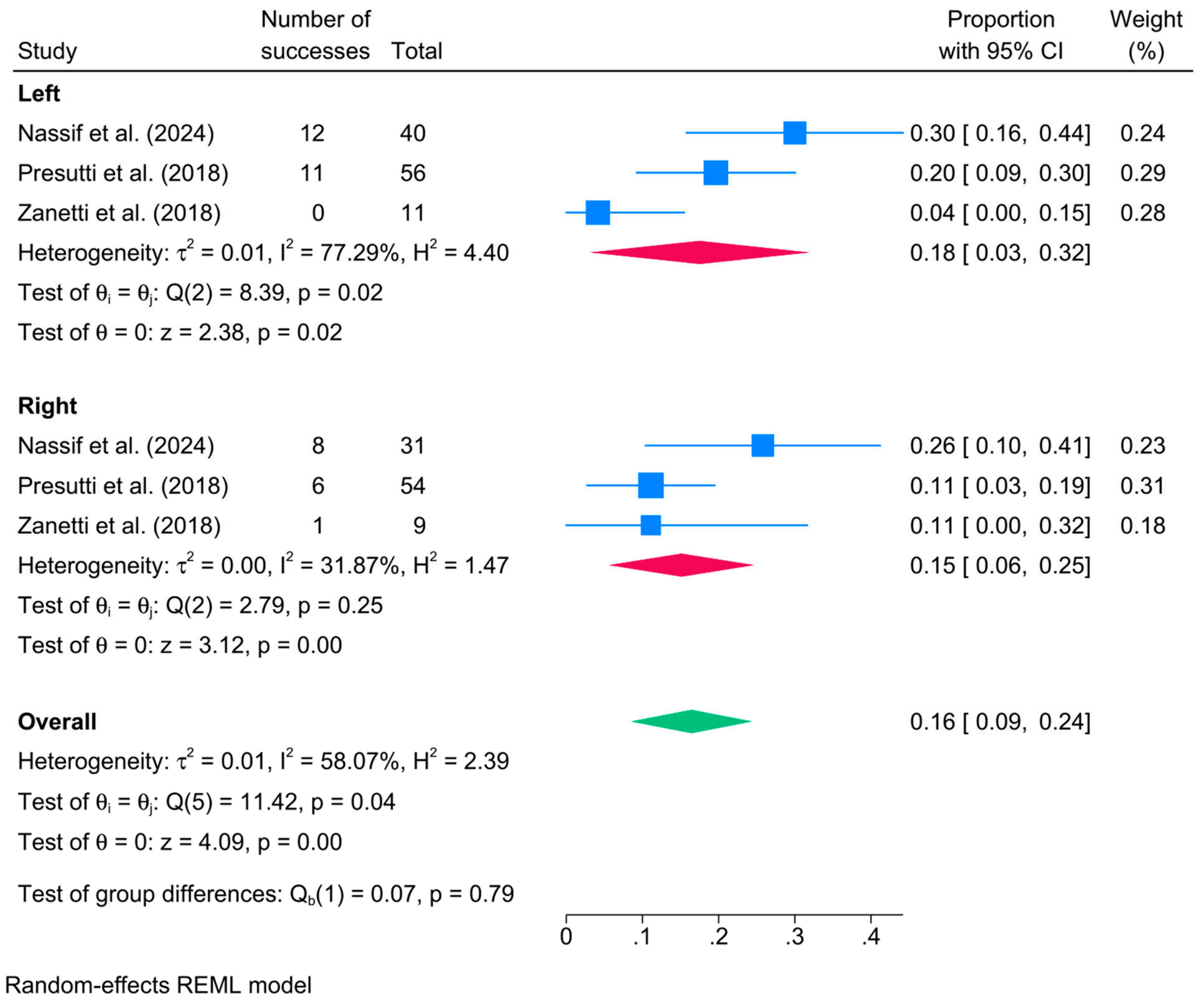

Figure S4. Forest plot showing the pooled cholesteatoma recurrence rate stratified by cholesteatoma laterality (left vs. right)

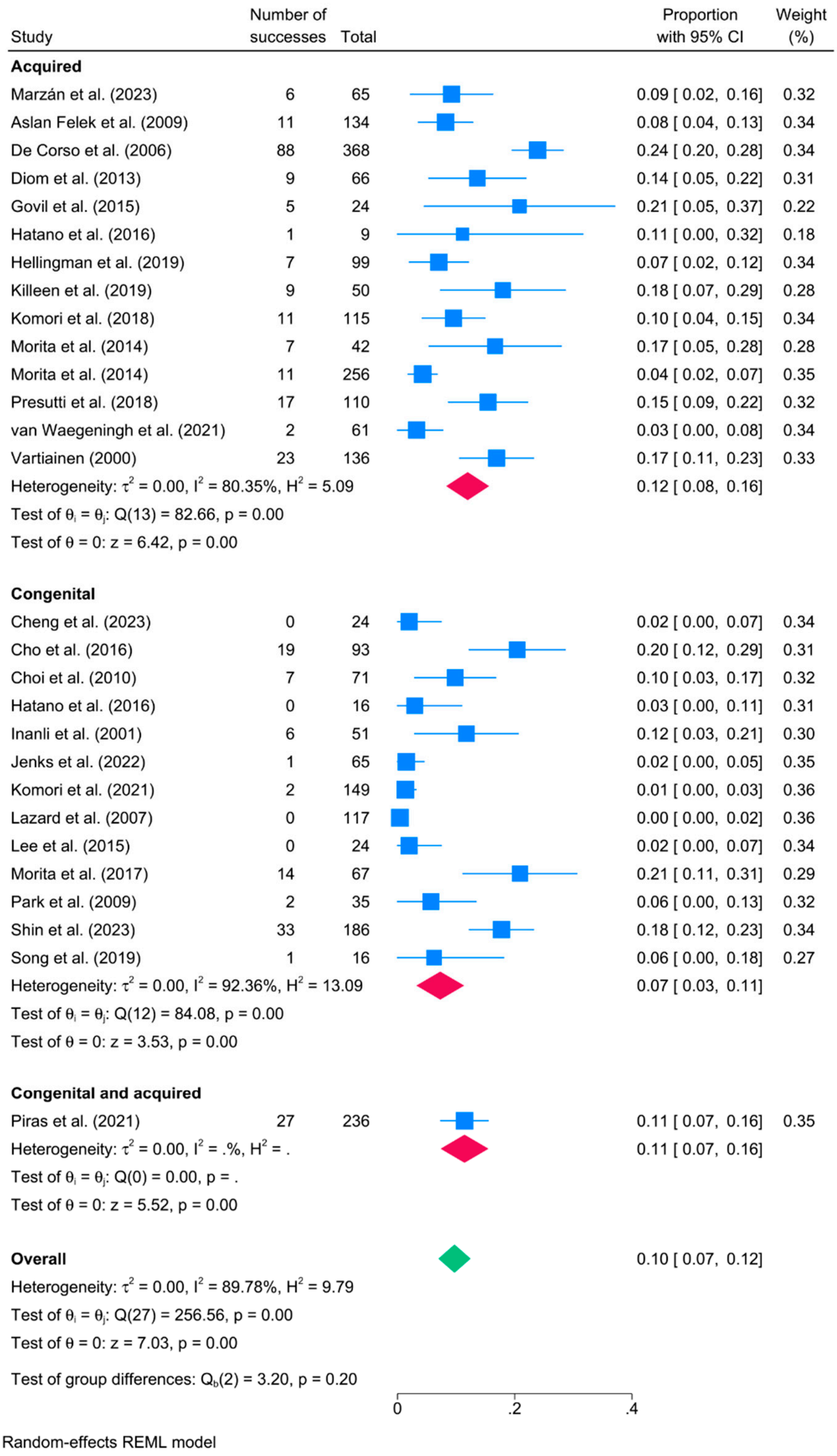

Figure S5. Forest plot showing the pooled cholesteatoma recurrence rate stratified by cholesteatoma type (acquired vs. congenital)

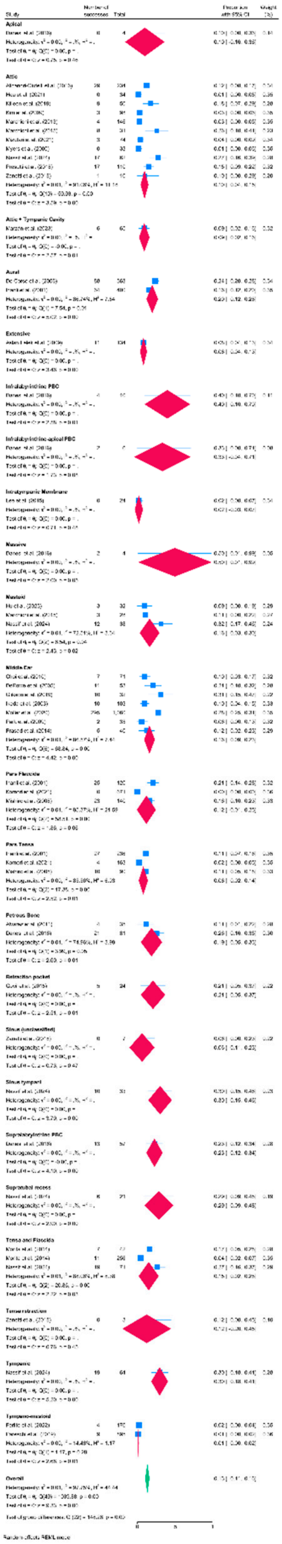

Figure S6. Forest plot showing the pooled cholesteatoma recurrence rate stratified by cholesteatoma location

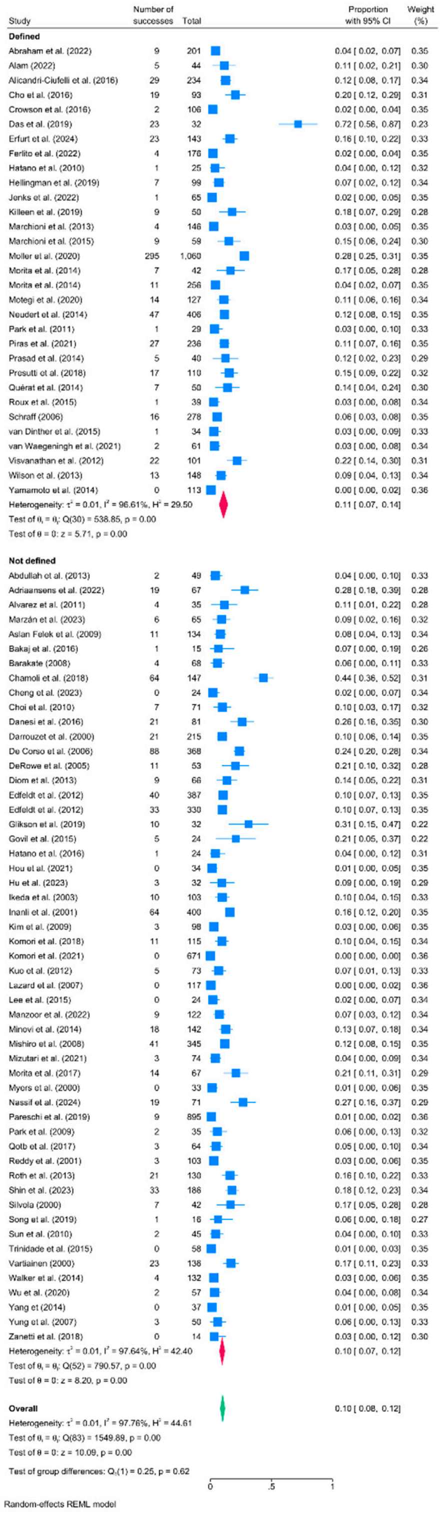

Figure S7. Forest plot showing the pooled cholesteatoma recurrence rate stratified by cholesteatoma recurrence definition

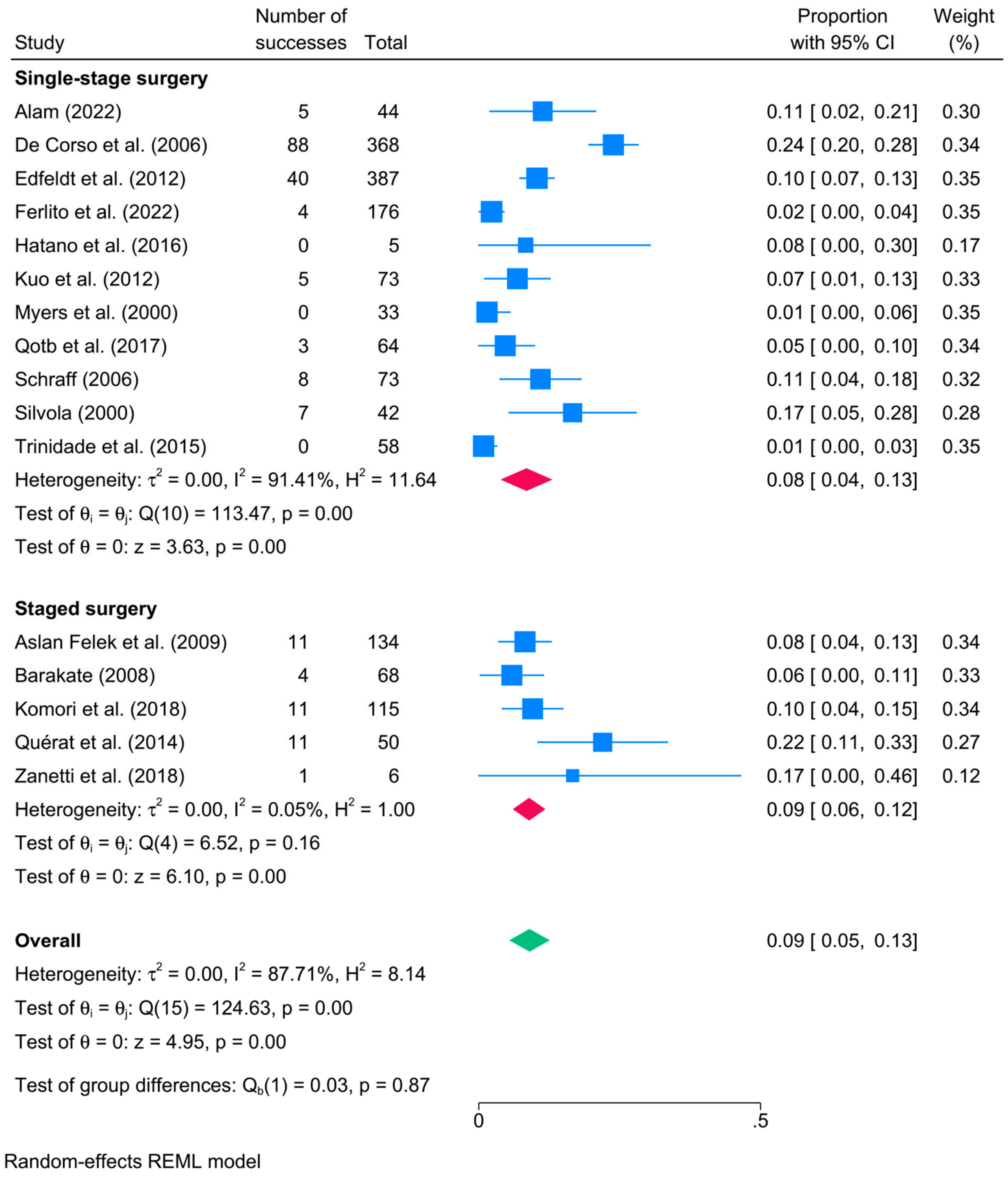

Figure S8. Forest plot showing the pooled cholesteatoma recurrence rate stratified by stage of surgery (single-stage vs. staged surgery)

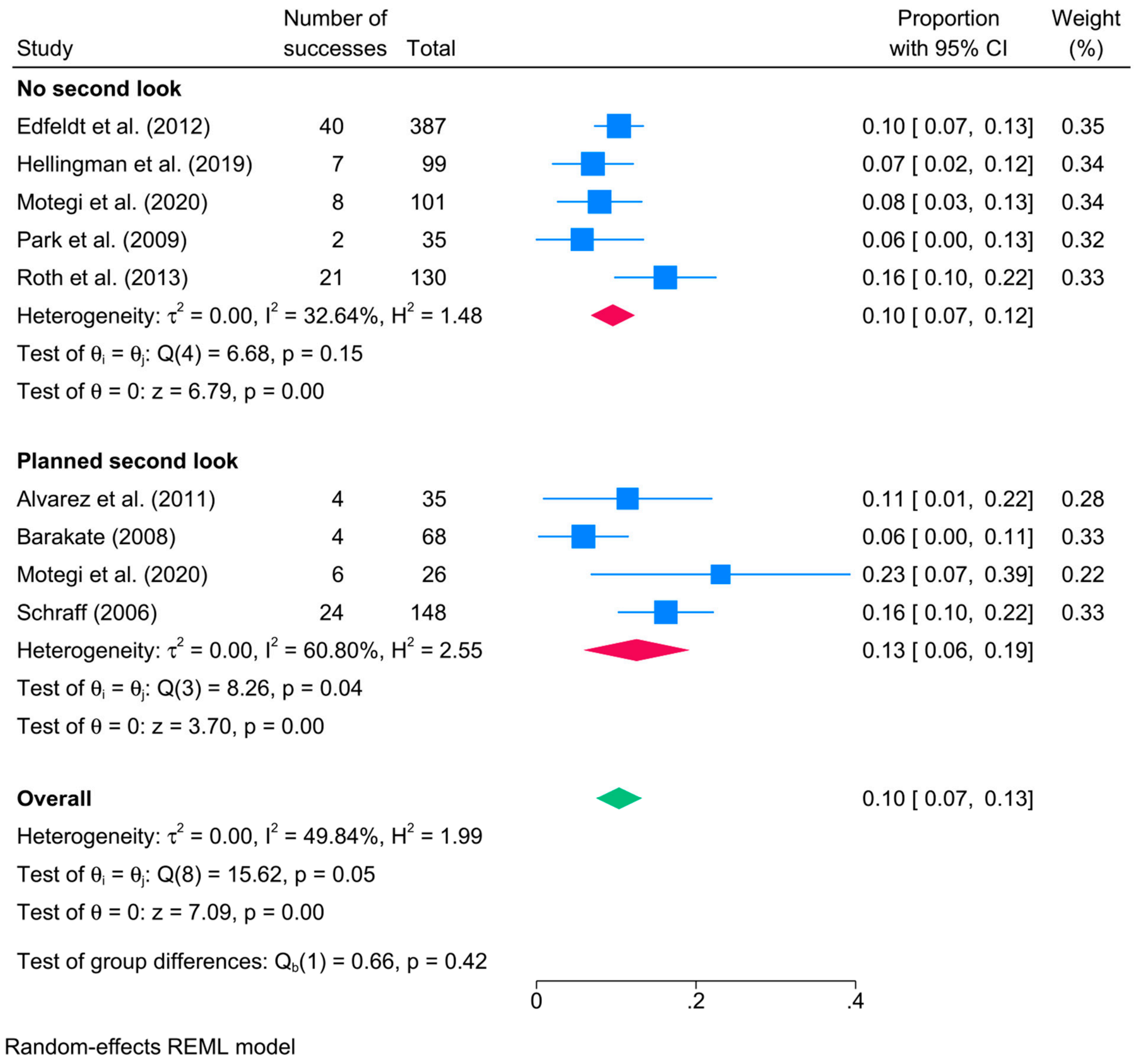

Figure S9. Forest plot showing the pooled cholesteatoma recurrence rate stratified by surgical intent (first look, second look surgery)

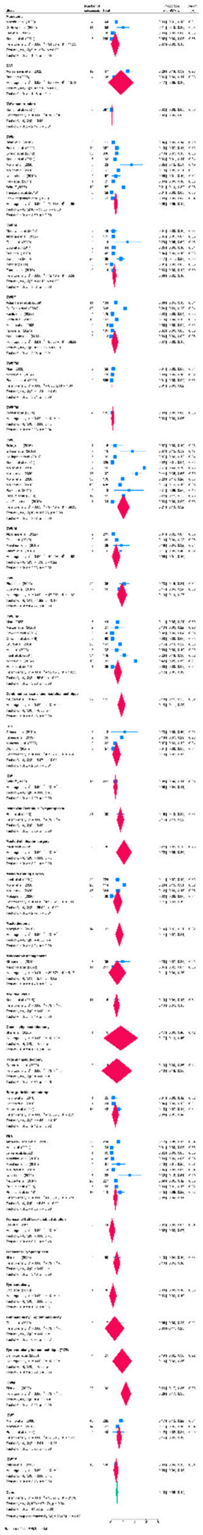

Figure S10. Forest plot showing the pooled cholesteatoma recurrence rate stratified by the type of surgical modality

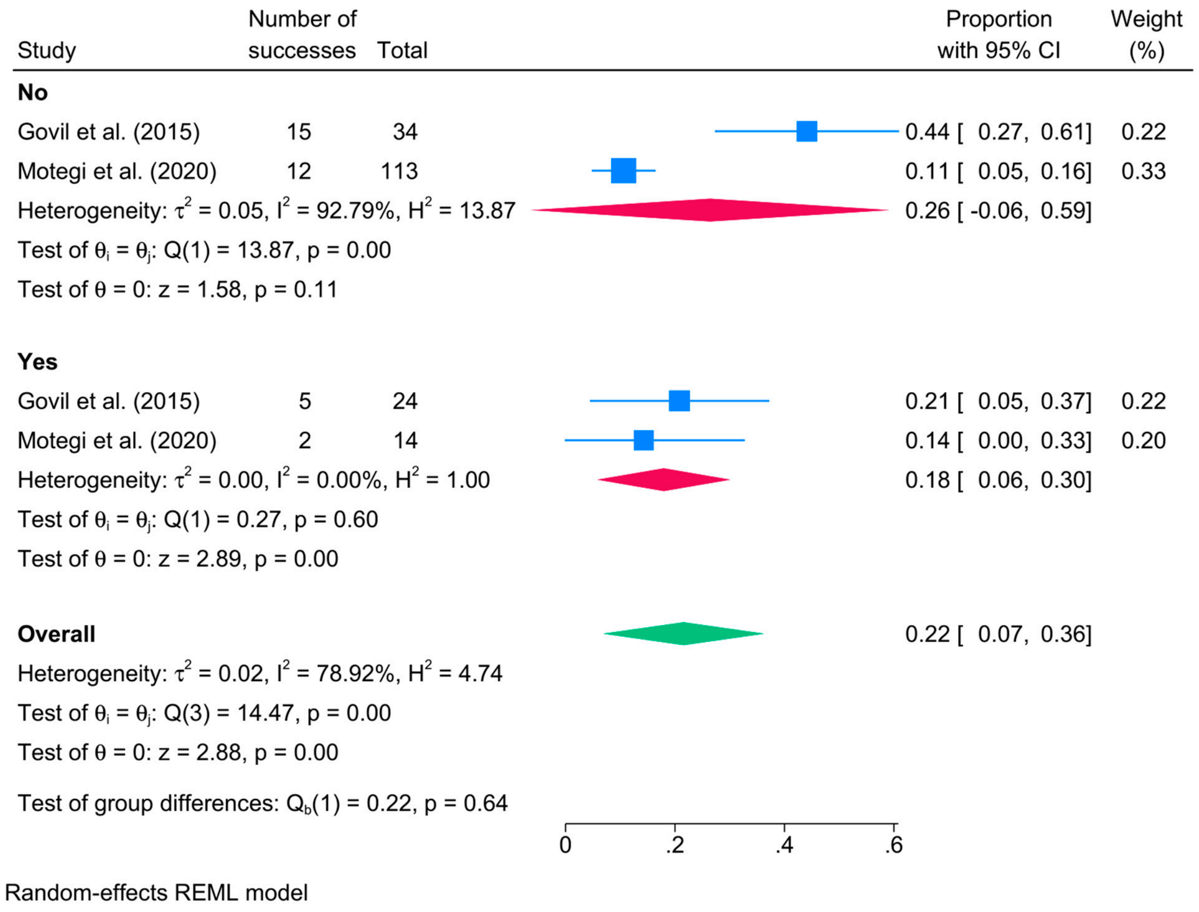

Figure S11. Forest plot showing the pooled cholesteatoma recurrence rate stratified by perioperative ventilation tube placement

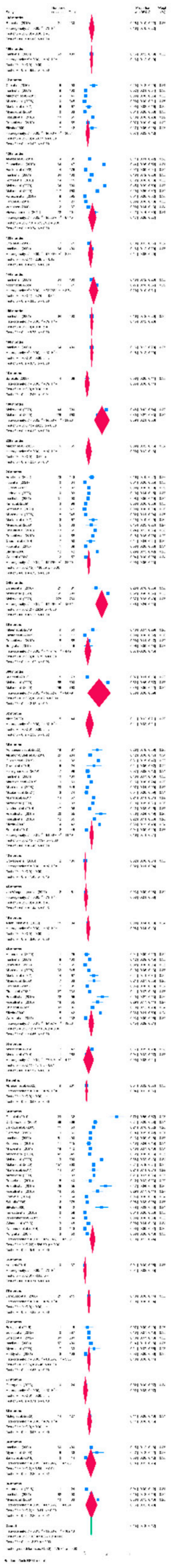

Figure S12. Forest plot showing the pooled cholesteatoma recurrence rate stratified by follow-up period
